# Impact of cladribine tablets on PROs in patients with MS: insights from the 1st interim analysis of the CLADFIT-MS study

**DOI:** 10.3389/fneur.2026.1765153

**Published:** 2026-04-10

**Authors:** Giovanna Borriello, Clara Grazia Chisari, Davide Maimone, Francesca Matta, Massimiliano Mirabella, Damiano Paolicelli, Francesco Assogna, Sandro Caradonna, Francesco Patti

**Affiliations:** 1 Multiple Sclerosis Center, San Pietro Fatebenefratelli Hospital, Rome, Italy; 2 Department of Public Health, Federico II University, Naples, Italy; 3 Department of Medical and Surgical Sciences and Advanced Technologies, GF Ingrassia, University of Catania, Catania, Italy; 4 UOS Multiple Sclerosis, AOU Policlinico "G Rodolico-San Marco", University of Catania, Catania, Italy; 5 Centro Sclerosi Multipla, UOC Neurologia, Azienda Ospedaliera per l'Emergenza Cannizzaro, Catania, Italy; 6 Centro Sclerosi Multipla, UOC Neurologia, Ospedale Garibaldi-Centro, Catania, Italy; 7 Multiple Sclerosis Center, Fondazione Policlinico Universitario "A. Gemelli" IRCCS, Rome, Italy; 8 Centro di Ricerca per la Sclerosi Multipla "Anna Paola Batocchi", Dipartimento di Neuroscienze, Università Cattolica del Sacro Cuore, Rome, Italy; 9 Neurology Unit, Department of Translational Biomedicine and Neuroscience (DiBraiN), Policlinico General Hospital, University of Study of Bari, Bari, Italy; 10 Merck Serono S.p.A., an Affiliate of Merck KGaA, Rome, Italy

**Keywords:** cladribine, highly active multiple sclerosis, Patient-Reported Outcomes, quality of life, wearable devices

## Abstract

**Background:**

Cladribine tablets are an oral short-course disease-modifying therapy (DMT) approved for the treatment of highly active relapsing multiple sclerosis (MS). While their efficacy has been demonstrated in clinical trials, limited real-world evidence is available on their impact on patient-reported outcomes (PROs) and the potential added value of wearable devices for continuous functional monitoring.

**Objectives:**

To evaluate the association between cladribine tablets use and PROs related to physical functioning and quality of life, and to explore the relationship between PROs and biometric data collected through wearable devices over 52 weeks in a real-world cohort of patients with highly active MS after therapeutic switch.

**Methods:**

CLADFIT-MS is a prospective, multicenter, observational phase IV study conducted in Italy. This interim analysis includes 190 patients with highly active MS who initiated cladribine tablets and had data available up to Week 52. PROs included the Multiple Sclerosis Impact Scale (MSIS-29), EuroQoL-5D-5L, and PROMIS-29 physical function and fatigue scores. Biometric data (e.g., range of movement, walking distance, sleep time) were collected using Fitbit® devices. Associations between PROs and wearable-derived data were analyzed using univariate mixed models and correlation matrices.

**Results:**

MSIS-29 physical scores and EQ-5D-5L remained stable over 52 weeks. PROMIS-29 fatigue scores showed slight improvement [from 54.6 (9.59) to 51.8 (10.30)], while PROMIS-29 physical function scores remained stable. An exploratory association was observed between baseline range of movement and changes in MSIS-29 scores (*p* = 0.0425), and between walking distance and PROMIS-29 fatigue (*p* = 0.0345). Biometric variables demonstrated moderate to strong inter-correlations, suggesting internal consistency of the wearable-derived data.

**Conclusion:**

In this real-world cohort of patients with highly active MS, cladribine tablets treatment was associated with the maintenance of PROs over one year. The integration of wearable technology provided objective metrics that were consistent with patient perceptions, supporting the feasibility of combining digital tools with PROs to monitor functional outcomes in MS clinical practice.

## Introduction

Multiple sclerosis (MS) is a chronic immune-mediated central nervous system disorder marked by neuroinflammation, demyelination, and progressive disability (1, 2). MS is also associated with cognitive, emotional, and functional impairments. Relapsing remitting MS (RRMS) accounts for about 85% of initial diagnoses (3, 4), with a subset experiencing highly active MS characterized by frequent relapses, rapid disability progression, and high lesion burden on Magnetic Resonance Imaging (MRI) (4, 5). Increasing evidence supports the paradigm of “time matters in MS,” whereby early treatment initiation is associated with better long-term outcomes and reduced progression of irreversible damage (5, 6, 7, 8, 9).

In a context in which the early use of high efficacy treatment is encouraged, the Guidelines from the European Committee for Treatment and Research in Multiple Sclerosis (ECTRIMS) and the European Academy of Neurology (EAN) recommend cladribine or other high-efficacy disease-modifying therapy (DMT) for patients with active relapsing MS, particularly in cases of suboptimal response to prior therapies or aggressive disease presentation (8, 10, 11).

Cladribine selectively targets lymphocyte subpopulations involved in MS pathogenesis by inducing apoptosis of B and T cells through intracellular phosphorylation and accumulation of active metabolites, while sparing innate immune cells (12). This results in a transient but profound reduction in adaptive immune cells, followed by gradual immune reconstitution, supporting durable disease control after short treatment courses (11).

Randomized clinical trials [CLARITY (10), CLARITY extension study (13), ORACLE (14)] and real-world studies demonstrate favorable outcomes with cladribine tablets in terms of relapse prevention, disability stabilization, treatment adherence, and manageable safety profiles (15–21).

To better explore the multidimensional nature of the MS burden and the efficacy of therapeutic interventions, traditional outcome measures can be complemented with Patient-Reported Outcome Measures (PROMs), which provide additional information on individual well-being as well as emotional and social life. The implementation of PROMs into clinical care and research enables a more patient-centered approach and can detect clinically significant treatment benefits even when traditional measures like Expanded Disability Status Scale (EDSS) remain unchanged (22–26).

A further step forward in the ever-improving understanding of the patient’s actual condition and the disease impact on patient’s functions is the recent availability of wearable devices that enable a continuous and objective monitoring of motor and non-motor symptoms, allowing detailed real time follow up beyond episodic clinical assessments (27–29). These devices effectively detect subtle functional changes, quantify motor disability, and capture symptom fluctuations such as fatigue (30, 31). Commercial devices like Fitbit® correlate with EDSS scores and disease-related physical limitations, while smartwatches and smartphones can monitor ambulatory impairment in natural environments (32–35). Wearables complement or predict clinical test results such as the 6-min walk test (29, 36).

This study aims to provide further evidence on the association between cladribine tablets use and Patient-Reported Outcomes (PROs) and their relationship with disability progression, using wearable devices to support continuous monitoring in a real-world setting for patients with highly active MS after therapeutic switch.

## Materials and methods

### Study design and setting

CLADFIT-MS is a prospective, observational, multicenter, phase IV study conducted in Italy across 29 referral centers for MS management. Data were collected in accordance with routine clinical practice and without any experimental intervention.

**Figure 1:**
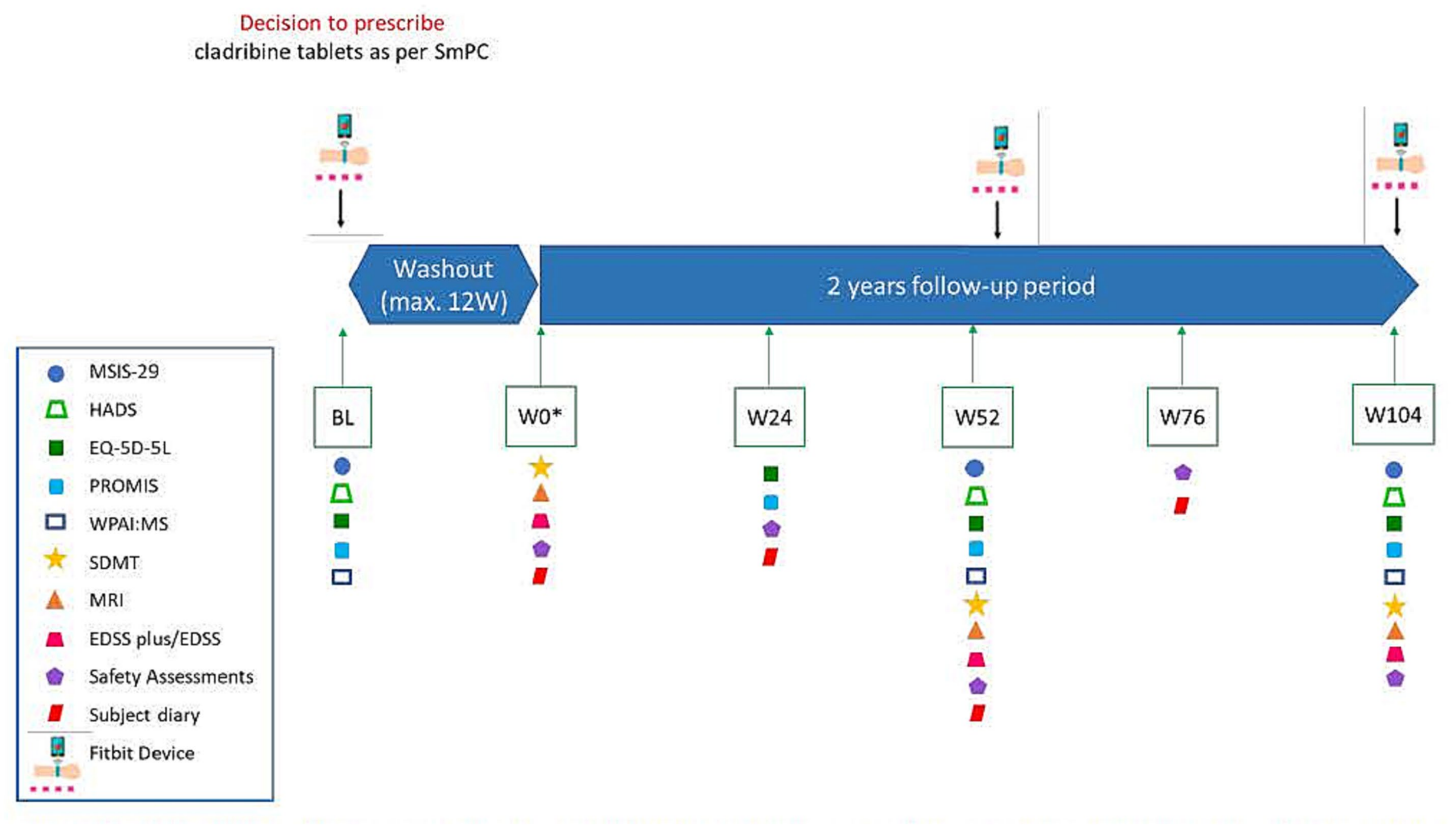
Study design. BL, baseline; EDSS, Expanded Disability Status Scale; EQ-5D-5L, EuroQoL 5-Dimension 5-Level; HADS, hospital anxiety and depression scale; MRI, Magnetic Resonance Imaging; MSIS-29, Multiple Sclerosis Impact Scale-29; PROMIS, Patient-Reported Outcomes Measurement Information System; SDMT, Symbol digit modalities test; SmPC, Summary of product characteristics; W, Week; WPAI:MS, work productivity and activity impairment, multiple sclerosis. *For subjects for whom a washout period is not needed, Baseline and Week 0 may be the same visit. If the period between Baseline and Week 0 is over 4 weeks, the MSIS-29 will be repeated at Week 0.

The baseline visit was scheduled at any time after the decision to initiate cladribine tablets treatment and before the beginning of the washout phase of the previous DMT. After baseline, the assessments were planned at the following timepoints: Week 0 (treatment initiation), Week 24, Week 52, Week 76, and Week 104. Wearable activity tracking using Fitbit® (Versa 3) devices was planned at baseline, Week 52 and Week 104. A diagram of the study design is displayed in Figure 1.

The detailed methods of CLADFIT-MS were previously published (37). This article reports the results of an interim analysis conducted on patients with available data up to Week 52. This interim analysis does not include the safety evaluation that will be conducted and included in the paper on the complete results.

### Study population

Eligible participants were adults (≥18 years) with a highly active RMS, defined as patients with one relapse in the previous year and at least one T1 gadolinium-enhancing (Gd+) lesion, or ≥ 9 T2 lesions while on therapy with other disease-modifying drugs (DMDs), or patients with ≥ 2 relapses in the previous year, whether on DMD treatment or not, switching to cladribine tablets as second-line treatment. Exclusion criteria included any contraindication to cladribine according to the product label, previous treatment with a second-line MS therapy, or any condition that, in the investigator’s judgment, would interfere with study participation or data interpretation. Inclusion and exclusion criteria are reported in the Supplementary Table 1.

### Ethics and informed consent

The study was conducted in accordance with the Declaration of Helsinki. Approval was obtained from local Ethics Committees at each participating site. All patients provided written informed consent before enrollment. The study protocol complies with the European Network of Centres for Pharmacoepidemiology and Pharmacovigilance (ENCePP) Checklist for Study Protocols (Revision 4). The study is registered in the European Union Electronic Register of Post-Authorization Studies (EU PAS) with the ID EUPAS43893.

### Study objectives and outcomes

The objective of the CLADFIT-MS study is to evaluate the association between cladribine tablets use and PROs, as well as the correlation of PROs with clinical and biometric parameters in patients with highly active RMS over a 104-week period.

The primary outcome measure of the CLADFIT-MS study is the change in the physical domain score of the Multiple Sclerosis Impact Scale (MSIS-29) at Week 52 compared to baseline.

In addition to the primary outcome, the change in the MSIS-29 physical domain score at Week 52 compared with baseline, this interim analysis focuses on the following subset of secondary outcome measures assessed at Week 52:

- Changes in EuroQoL 5 Dimension 5 Level (EQ-5D-5L) index score compared to baseline;
- Changes in Patient-Reported Outcomes Measurement Information System [PROMIS-29 (38)] physical function compared to baseline;
- Association between changes in PROs (MSIS-29 and PROMIS-29 fatigue) and data from wearable activity trackers (i.e., Fitbit® - range of movement, step counts, walking speed, heart rate, burned calories and sleeping time).

Pairwise Pearson correlation coefficients were also calculated among all biometric parameters measured by the Fitbit® device: range of movements (steps), walking distance (meters), walking speed (m/s), calories burned (kcal), heart rate (bpm), and sleep duration (hours), to evaluate the interrelationships between these variables.

The publication of the final results of the study will report on the outcome measures, such as EDSS and MRI clinical assessments, that are planned in the study protocol but are not the object of this interim analysis.

### Wearable activity tracking (Fitbit®)

Fitbit® wearables devices were used to capture real-world mobility and physiological signals at three standardized windows aligned with study visits: the two days immediately after baseline, and the two days preceding Weeks 52 and 104 (six prespecified days total). Limiting analysis to these windows, even though participants may wear devices more frequently, minimizes selection bias and ensures comparability across time points. The protocol balances participant burden with data quality by combining hourly granularity (steps, calories, average heart rate) and daily granularity (walking distance, walking speed, sleep duration, device wear time and maximum wear time). From these inputs, meaningful 2-day aggregates per visit window were derived - total steps, total calories, mean hourly heart rate, total sleep hours, total walking distance, mean walking speed - and quantify non-wear (hours) as the difference between maximum wear time and device wear time.

Analytically, these derived variables provide complementary perspectives on function and behavior: steps and distance reflect ambulatory activity; walking speed approximates habitual pace; heart rate and calories capture physiological effort; sleep hours inform rest and recovery; and non-wear time supports compliance and quality control. Analysis has been focused on within-participant change relative to baseline across the three windows, reporting means and 95% confidence intervals (CIs). To preserve interpretability, consistent preprocessing was applied (e.g., excluding non-wear segments, using the prespecified windows), and sensitivity analyses were considered adjusting for potential temporal confounders and handling missingness with predefined rules. This approach aims to provide stable, comparable snapshots of activity and physiology that can be interpreted alongside clinical outcomes and other PROs.

Wearable-derived outcomes were analyzed in a pre-specified, standardized 2-day window at each timepoint and are intended as exploratory measures. Given limited sample sizes for complete wearable data and multiple comparisons, associations are hypothesis-generating and should be interpreted cautiously.

### Statistical analysis

Sample size for the study was determined based on the primary outcome measure. Assuming a standard deviation (SD) of 13 and aiming for a two-sided 95% CI with a maximum width of 4, a total of 193 evaluable patients were required. Accounting for a potential dropout rate of 10%, the planned sample size was set at 215 patients.

Data analysis was conducted using Statistical Analysis System (SAS) Version 9.4. Analyses were primarily descriptive. For exploratory association analyses between changes in PROs and wearable-derived parameters, univariate mixed models were fitted and *p*-values were computed; however, these p-values are presented for exploratory purposes only. No adjustment for multiple comparisons was applied; therefore, findings should be interpreted cautiously, with emphasis placed on effect estimates and 95% CIs.

Continuous variables were summarized using mean, SD, median, range, and interquartile range (IQR); categorical variables by counts and percentages. Missing data were not imputed.

The results of the primary outcome measure MSIS-29 physical domain score were summarized with mean value with the corresponding 95% CI. In addition to descriptive summaries, a mixed model for repeated measures (MMRM) sensitivity analysis was performed for the primary outcome to assess robustness to missing follow-up data. The dependent variable was change from baseline in MSIS-29 physical domain score, and the model included a fixed categorical effect of visit (Week 52) and the following baseline covariates: baseline MSIS-29 physical domain score, age category (≤40 vs. > 40 years), gender, baseline EDSS category (0, 1–3.5, 4–10), disease duration category (<24 vs. ≥ 24 months), employment status (employed vs. not employed), and relapse activity in the previous year (≤1 vs. > 1). Degrees of freedom were estimated using the Kenward-Roger approximation. The MMRM included all evaluable patients with baseline and at least one post-baseline MSIS-29 physical domain value; missing data were not imputed.

### Descriptive statistics were provided for EQ-5D-5L and PROMIS-29 score

Univariate mixed models were used to assess the associations between changes in PROs (MSIS-29 physical domain score and PROMIS-29 fatigue) and Fitbit® biometric parameters. Mixed models with the change from baseline of MSIS-29 physical domain scores and PROMIS-29 fatigue scores as the dependent variables were fitted. The covariates of these models were the baseline values of the dependent variable, change from baseline values at Week 52 of parameter evaluated from wearable activity tracker and the interaction between them. Degrees of freedom were estimated using the Kenward-Roger Degrees of Freedom Approximation. *p*-values and CIs were calculated with a t statistic using sum of squares from the model.

In addition, a correlation matrix was computed to explore the relationships among biometric parameters collected via wearable activity trackers. For each pair of variables, a correlation coefficient was computed, ranging from −1.0 (i.e., perfect negative correlation) to 1.0 (i.e., perfect positive correlation). In the resulting correlation matrix, the main diagonal is always 1.0, reflecting perfect self-correlation. The matrix is symmetrical, with mirrored values above and below the diagonal.

## Results

### Patient disposition

Patient disposition data refer to the cut-off date of January 23, 2024, and are not final. Of the 228 patients who signed the informed consent form (ICF), 190 received at least one dose of cladribine tablets and were included in the Full Analysis Set (FAS). The Safety Analysis Set, which also included 190 patients, and the Treatment Completer Analysis Set, comprising 94 patients who had completed the full course of cladribine tablets treatment (i.e., two treatment weeks with 4–5 doses per week in both Year 1 and Year 2), will be described in detail in the final 104-week analysis.

### Baseline characteristics of the analyzed population

#### Demography

The majority of patients were female (70.5%) and 62.6% were aged 40 years or younger. Most patients (77.4%) had received an MS diagnosis more than two years before study entry. At baseline, over 60% of patients were employed (61.1%). Other demographics and baseline characteristics are reported in Table 1.

**Table 1:** Demographics and baseline characteristics.

Population	Data
Parameter	*N* = 190
Gender, *n* (%)	
Male	56 (29.5)
Female	134 (70.5)
Age at ICF obtainment (years)	
Mean (SD)	38 (10.5)
Median	37
Q1; Q3	29; 46
Age categories, *n* (%)	
≤ 40 years	119 (62.6)
> 40 years	71 (37.4)
Disease duration, *n* (%)	
MS diagnosis < 24 months	43 (22.6)
MS diagnosis ≥ 24 months	147 (77.4)
Employment status at baseline, *n* (%)	
Employed	116 (61.1)
Not employed	72 (37.9)
Missing	2 (1.1)
Baseline height (cm)	
*N* (%)	150 (78.9)
Missing, *n* (%)	40 (21.1)
Mean (SD)	166.3 (8.90)
Median	165.0
Q1; Q3	160.0; 171.0
Baseline weight (kg)	
*N* (%)	187 (98.4)
Missing, *n* (%)	3 (1.6)
Mean (SD)	69.5 (16.46)
Median	66.0
Q1; Q3	57.0; 80.0

ICF, informed consent form; MS, multiple sclerosis; Q1, Q3, interquartile range; SD, standard deviation.

#### Disease history

The mean time since diagnosis (SD) was 6.7 (6.07) years, and most patients had presented with sensory (45.8%) or visual symptoms (36.3%) after their first MS attack. Nearly all patients (99.5%) fulfilled the McDonald 2017 criteria. Most patients (71.6%) had a baseline EDSS score between 1.0 and 3.5 (Table 2).

**Table 2:** Disease history.

Population	Data
Parameter	*N* = 190
Elapsed time since diagnosis (years)	
Mean (SD)	6.7 (6.07)
Median	4.7
Q1; Q3	2.2; 9.2
Major systems affected after first attack, *n* (%)	
Pyramidal function	42 (22.1)
Cerebellar function	12 (6.3)
Brain stem function	35 (18.4)
Sensory function	87 (45.8)
Bowel and bladder function	8 (4.2)
Visual (optic) function	69 (36.3)
Cerebral (mental) function	8 (4.2)
Subjects fulfilling McDonald 2017 criteria, *n* (%)	
Yes	189 (99.5)
No	1 (0.5)
Elapsed time since first symptoms (years)	
*N* (%)	189 (99.5)
Missing, *n* (%)	1 (0.5)
Mean (SD)	8.1 (6.68)
Median	6.2
Q1; Q3	3.2; 10.8
EDSS at baseline, *n* (%)	
0	14 (7.4)
1–3.5	136 (71.6)
4–10	10 (5.3)
Missing	30 (15.8)

EDSS, expanded disability status scale; Q1, Q3, interquartile range; SD, standard deviation.

In the 12 months prior to treatment initiation, 83.2% of patients experienced a number of relapses ≤ 1. The mean relapse duration (SD) was 21.7 (32.87) days (Table 3). Sensory and pyramidal functions were the most frequently affected during relapses (53.7 and 24.7%, respectively). In half of the cases, relapses were treated with corticosteroids, while hospitalizations were rare (2.6%). Supplementary Table 2 provides additional details on relapses characteristics.

**Table 3:** Disease history – relapse.

Population	Data
Parameter	*N* = 190
Relapses within 12 months prior to baseline, *n* (%)	
0	15 (7.9)
1	143 (75.3)
2	27 (14.2)
3	3 (1.6)
≥ 4	2 (1.1)
Relapses in the last year before cladribine, *n* (%)	
≤ 1	158 (83.2)
> 1	32 (16.8)
Estimated duration of the relapse (days)	
*N* (%)	183 (96.3)
Missing, *n* (%)	7 (3.7)
Mean (SD)	21.7 (32.87)
Median	13.0
Q1; Q3	0.0; 28.0

Q1, Q3, interquartile range; SD, standard deviation.

### Primary outcome

#### MSIS-29 physical domain

MSIS-29 is a 29-item PRO instrument comprising a 20-item physical scale and a 9-item psychological scale. The 20 items of the MSIS-29 physical domain are scored on a 1–5 Likert scale; summed raw scores are transformed to a 0–100 metric in which higher values indicate greater MS impact (worse health), so decreases denote improvement (39). In the population with data at both baseline and Week 52 (*n* = 157), the mean MSIS-29 physical domain score (SD) was 13.4 (14.21) at baseline and 12.8 (15.68) at Week 52, with a small, non-significant mean (SD) change of 0.1 (9.10) (*p* = 0.8703; Table 4), indicating stable scores over 52 weeks. Additional information on MSIS-29 physical domain scores and assessment availability at baseline and Week 52 is provided in Supplementary Table 3.

**Table 4:** Change in the MSIS-29 physical domain score at week 52 compared with baseline.

Parameter	Baseline	Week 52	Change from baseline*
*N* (%)	186 (97.9%)	161 (84.7%)	157 (82.6%)
Missing, *N* (%)	4 (2.1%)	29 (15.3%)	33 (17.4%)
Mean (SD)	13.4 (14.21)	12.8 (15.68)	0.1 (9.10)
95% CI (LCL; UCL)	11.3; 15.4	10.4; 15.3	−1.3; 1.6
Median	8.8	6.3	0.0
Q1; Q3	2.5; 20.0	1.3; 17.5	−5.0; 3.8

*Change from baseline included only those patients with both a baseline and week 52 value.

CI, confidence interval; LCL, lower confidence limit; Q1; Q3, interquartile Range; SD, standard deviation; UCL, upper confidence limit.

As a sensitivity analysis addressing missing follow-up data, an MMRM was conducted in the FAS including all patients with baseline and at least one post-baseline MSIS-29 assessment (160/190 evaluable patients). The estimated Least Squares (LS)-mean change from baseline to Week 52 was 2.6 [Standard Error (SE): 1.46; 95% CI: −0.31; 5.49] (Table 5).

**Table 5:** Sensitivity analysis: MMRM analysis of changes in MSIS-29 physical domain scores using baseline variables as covariates.

Change from baseline of MSIS-29 physical domain scores	Estimate	Standard error	Degrees of freedom	*p*-value	95% CI
Number of evaluable patients*, *n* (%)	160 (84.2)				
Number of patients with change from baseline at week 52, *n* (%)	157 (82.6)				
Estimated LS-means for change from baseline to week 52	2.6	1.46	118.1	0.0790	−0.31; 5.49
Estimated effects Intercept	−6.2	4.12	46.5	0.1406	
Visit week 52	3.5	3.16	16.2	0.2899	
Baseline score	−0.3	0.07	117.4	<0.0001	
Age					
> 40 years	2.6	1.63	119.2	0.1090	
Gender					
Female	1.4	1.63	118.7	0.4020	
EDSS at baseline					
1–3.5	3.1	2.39	118.8	0.1938	
4–10	14.2	4.20	116.7	0.0010	
Disease duration < 24 months	2.1	1.85	117.8	0.2554	
Employment status Not employed	1.5	1.61	119.0	0.3443	
Relapses in the previous year > 1	0.4	1.96	121.1	0.8372	

*The evaluable patients were patients with a baseline and at least one post-baseline value of the dependent variable.

CI, confidence interval; EDSS, expanded disability status scale; LS, least squares; MMRM, mixed model repeated measures; MSIS-29, multiple sclerosis impact scale-29.

### Secondary outcomes

#### EQ-5D-5L

The EQ-5D-5L captures five dimensions (mobility, self-care, usual activities, pain/discomfort, anxiety/depression) with five response levels that form a 5-digit health state, which is converted to a country-specific utility index anchored at 1 (full health) and 0 (dead), with values <0 indicating states worse than dead. In patients with available data at baseline and Week 52 (N = 158), the mean EQ-5D-5L index score (SD) remained stable, changing from 0.782 (0.1844) to 0.780 (0.2099), with a mean difference of −0.011 (0.1492) (Table 6).

**Table 6:** EQ-5D-5L index score.

Parameter	Baseline	Week 52	Change from baseline*
*N* (%)	185 (97.4)	162 (85.3)	158 (83.2)
Missing, *n* (%)	5 (2.6)	28 (14.7)	32 (16.8)
Mean (SD)	0.782 (0.1844)	0.780 (0.2099)	−0.011 (0.1492)
95% CI (LCL; UCL)	0.7552; 0.8087	0.7471;0.8122	−0.0346; 0.0123
Median	0.768	0.802	0.0
Q1; Q3	0.679; 1.000	0.679; 1.000	−0.094; 0.025

^*^Change from baseline included only those patients with both a baseline and week 52 value.

CI, confidence interval; LCL, lower confidence limit; Q1; Q3, interquartile range; SD, standard deviation; UCL, upper confidence limit.

#### PROMIS-29 physical function

PROMIS-29 physical function is scored on the PROMIS T-score metric standardized to a general-population mean (SD) of 50 (10), with higher T-scores representing better physical function (e.g., 60 ≈ +1 SD). At Week 52, the mean change (SD) from baseline in PROMIS-29 physical function scores was −0.1 (6.37) in the 162 patients with available data, with mean score (SD) of 50.3 (9.11) at baseline and 50.7 (10.45) at Week 52 (Table 7).

**Table 7:** PROMIS-29 physical function.

Parameter	Baseline	Week 52	Change from baseline*
*N* (%)	184 (96.8)	162 (85.3)	162 (85.3)
Missing, *n* (%)	6 (3.2)	28 (14.7)	28 (14.7)
Mean (SD)	50.3 (9.11)	50.7 (10.45)	−0.1 (6.37)
95% CI (LCL; UCL)	49.0; 51.7	49.1;52.3	−1.1;0.9
Median	49.4	51.2	0.0
Q1; Q3	43.8: 57.8	44.1; 63.4	−4.1; 3.4

^*^Change from baseline included only those patients with both a baseline and week 52 value.

CI, confidence interval; LCL, lower confidence limit; Q1; Q3, interquartile range; SD, standard deviation; UCL, upper confidence limit.

### Association between changes in PROs (MSIS-29 and PROMIS-29 fatigue) and data from Fitbit®

#### MSIS-29

**Figure 2:**
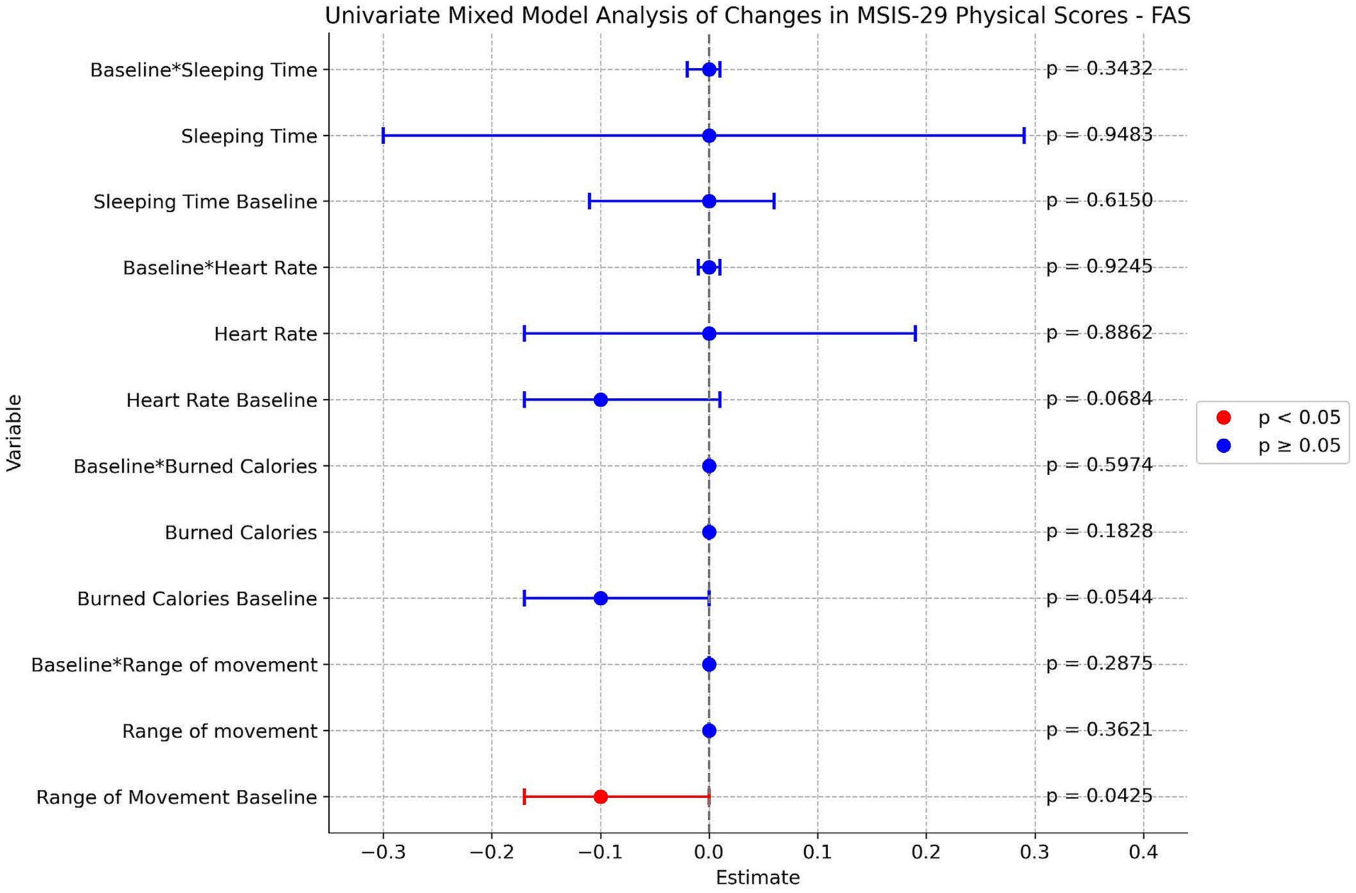
Univariate mixed model analysis of changes in MSIS-29 physical domain score.

An exploratory association (*p* = 0.0425) was observed between the baseline range of movement derived from Fitbit® and the change in MSIS-29 physical domain score at Week 52. Specifically, higher baseline levels of physical activity were associated with a reduction in self-reported physical impact over time (Figure 2 and Supplementary Table 4).

#### PROMIS-29 fatigue

**Figure 3:**
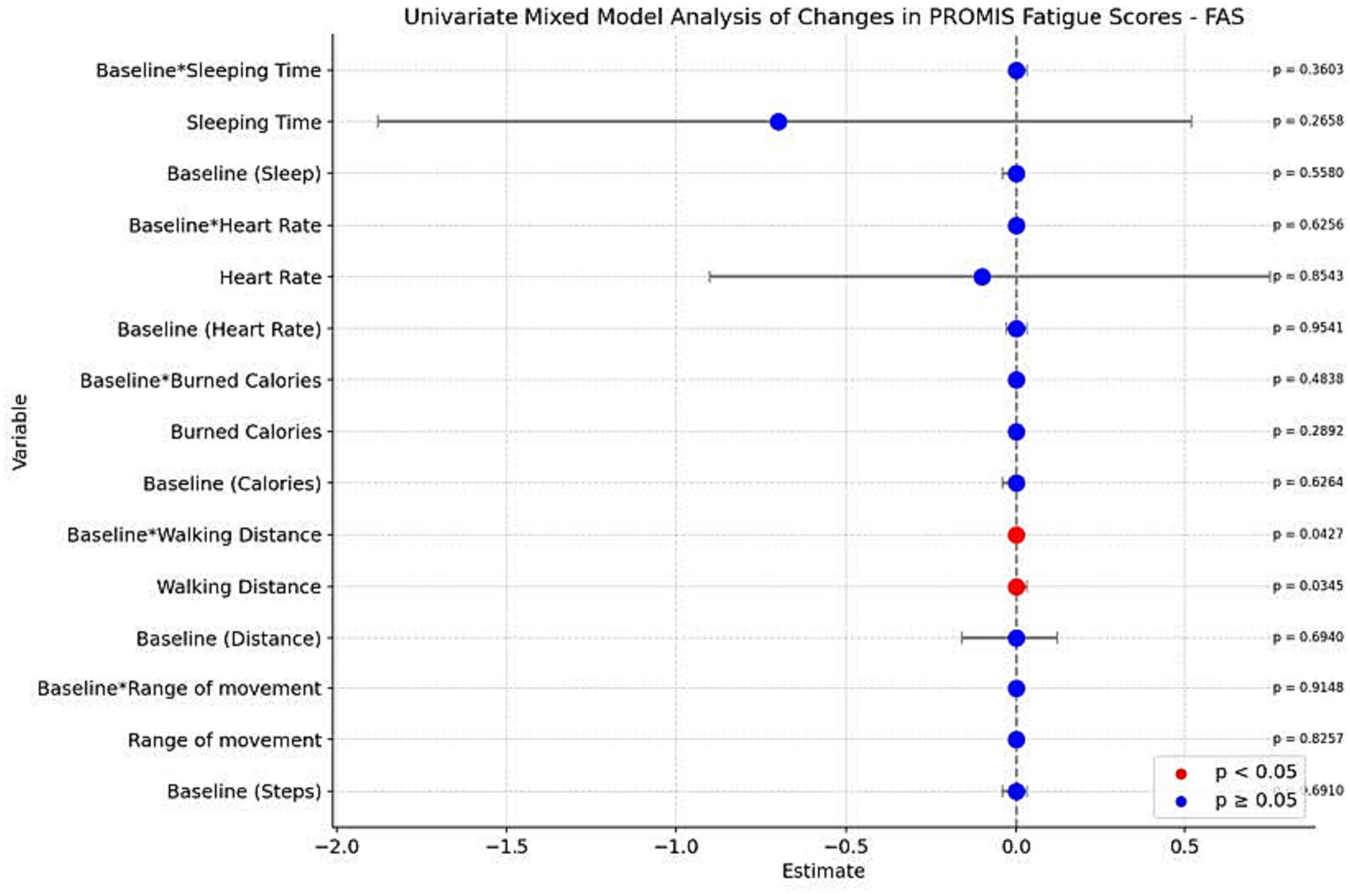
Univariate mixed model analysis of changes in PROMIS-29 fatigue scores.

An increase in walking distance was associated with increased PROMIS-29 fatigue scores (*p* = 0.0345), although this relationship was moderated by baseline fatigue levels, showing an inverse interaction (*p* = 0.0427). No other biometric parameters were significantly associated with changes in fatigue scores (Figure 3).

### Correlation between biometric parameters

Data on biometric parameters collected at baseline and Week 52 and change from baseline are reported in Supplementary Table 5. Additional information on the availability of wearable-derived parameters at baseline and Week 52 is provided in Supplementary Table 6. A summary of the effective sample size contributing to each wearable-derived outcome at baseline, Week 52, and for the change from baseline analyses is presented in Supplementary Table 7.

**Figure 4:**
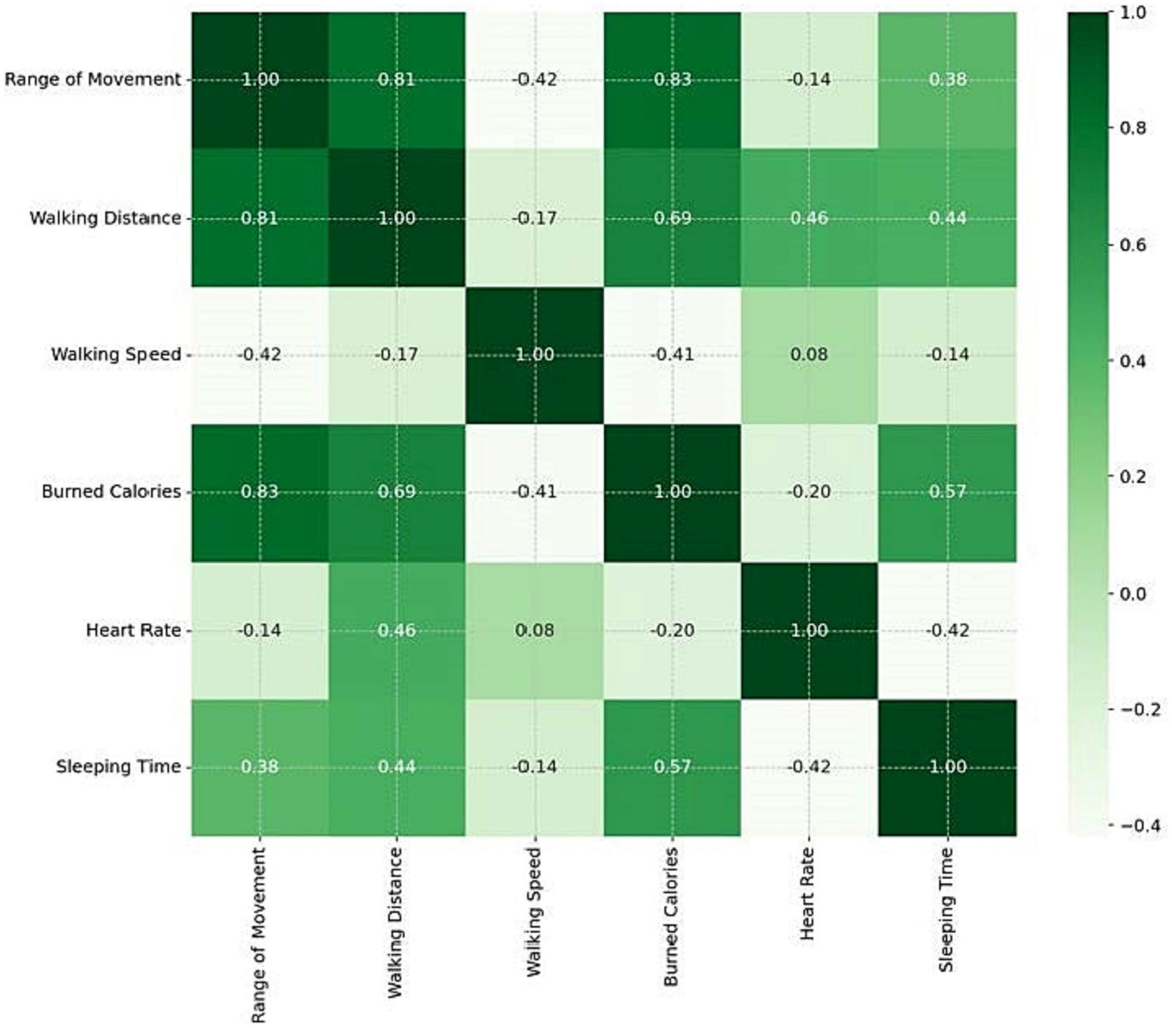
Correlation analysis between Fitbit® devices parameters.

Correlation analysis between biometric variables collected via Fitbit® in the study population revealed a strong direct correlation between range of movement, walking distance (r = 0.81) and burned calories (r = 0.83). Moderate direct correlations were also observed between walking distance and burned calories (r = 0.69) and between burned calories and sleeping time (r = 0.57). Walking speed showed a moderate inverse correlation with range of movement (r = −0.42). No strong correlations were found for heart rate (Figure 4).

## Discussion

This interim analysis of the CLADFIT-MS study describes the evolution of PROs and biometric parameters during the first year of treatment with cladribine tablets in a real-world cohort of patients with highly active MS.

Concerning the primary outcome, although the change in MSIS-29 physical domain was not statistically significant at Week 52, the overall stability of the score over one year in a highly active RMS population suggests a maintenance of perceived physical function. It is important to consider that these results are derived from a pre-planned interim analysis. The full 104-week follow-up may provide additional insights into the long-term evolution of patient-reported physical impact and the potential delayed benefits of cladribine tablets treatment.

In this interim analysis, PROs related to physical function and quality of life remained stable throughout the 52-week observation period suggesting a preservation of patients perceived functional status and quality of life during cladribine tablets therapy.

In the phase IV, open-label, prospective CLARIFY study (40), statistically significant improvements were observed in the least square mean MS quality of life (MSQoL)-54 overall QoL scores, which increased by 2.71 points (95% CI: 1.32 to 4.10; *p* = 0.0001) and 2.88 points (95% CI: 1.41 to 4.34; p = 0.0001) at Months 12 and 24, respectively, compared with baseline. The significant improvement in HRQoL observed at Year 2 in CLARIFY-MS was sustained over the subsequent 2-year CLARIFY-MS EXT study (41), with least-squares mean increases at Month 48 of 3.69 points (95% CI 1.71–5.67; *p* = 0.0003) in the MSQoL-54 physical health composite and 5.13 points (2.73–7.53; *p* < 0.0001) in the Mental Health composite. The final 104-week analysis of our study will provide a more complete picture and will allow us to determine whether the results are consistent with the improvements observed in the CLARIFY study.

The stability of PROs over 12 months suggests preservation of patients perceived functional status and quality of life under cladribine tablets therapy, which appears to be associated with the maintenance of subjective well-being.

The wearable-derived analyses were exploratory and performed without adjustment for multiplicity; therefore, the observed associations should be considered as hypothesis-generating. An exploratory association between the baseline range of movement, as recorded by Fitbit®, and the reduction of MSIS-29 physical domain scores at Week 52 was observed, but its significance is limited by the small number of observations. The observed association between the increase of the walking distance recorded by Fitbit® data and the increase in the PROMIS-29 fatigue scores is to be interpreted with caution. Given the short 2-day sampling windows, missingness, lack of multiplicity adjustment, and the presence of an interaction with baseline fatigue, this finding may reflect variability and/or residual confounding rather than a direct relationship. Confirmation will be sought in the final 104-week analysis.

The results upon completion of the 104-week observation period will allow us to determine the consistence with previous studies that explored the integration of wearable technologies into MS research and clinical practice (28, 31, 32, 34, 42–47). Because this interim analysis was restricted to patients with available assessments at Week 52 (e.g., MSIS-29 physical domain paired baseline/Week-52 data in 157/190 patients), results may be affected by selection bias if patients with missing follow-up data differed systematically from those with complete data. Missing data were not imputed, and wearable-derived parameters were available only in a subset of patients, raising the possibility of informative missingness (e.g., patients with worse functional status or lower device adherence may be under-represented). These factors may lead to an overestimation of stability in PROs and limit inference. Sensitivity analyses will be considered in the final 104-week analysis to evaluate robustness of findings. Due to the preliminary nature of this interim analysis, complete 52-week clinical context data (relapses, discontinuations/switches, tolerability) are not yet available at this stage and will be addressed in the final publication reporting the complete study results. Furthermore, given the observational design of the study and the interim nature of this analysis, the findings, while suggestive of an association between treatment and PRO stability, do not allow causal inference and do not exclude the potential contribution of other factors (e.g., regression to the mean, post-relapse recovery, DMT switching) to the observed stability of PROs. Although the sample size was planned to achieve a precision target for the primary outcome (193 evaluable; 215 planned), analyses in this interim report were descriptive and exploratory; accordingly, no *a priori* power calculation to detect a prespecified effect size was implemented, limiting inference regarding the magnitude of change. The innovative aspect and key strength are the integration of validated PROs and wearable-derived data in a routine clinical setting.

In conclusion, patients treated with cladribine tablets showed stability in self-reported physical function and quality of life after one year of treatment. The treatment with cladribine tablets appears to be associated with stability of PROs and biometric parameters, and this evidence is even more noticeable considering that the study population is composed of patients with highly active MS. The overall results of the CLADFIT-MS study with data up to Week 104 will be reported upon study completion and will provide a comprehensive assessment outcomes after two years of therapy, including the correlation between PROMs and the evolution of clinical measures. Additional future studies will hopefully provide insights into longer-term outcomes beyond the two-year follow-up.

## Data Availability

The data analyzed in this study is subject to the following licenses/restrictions: the datasets generated for this study are available from the Corresponding Author upon reasonable request. Requests to access these datasets should be directed to Francesco Patti, francesco.patti@unict.it.

## The CLADFIT-MS investigators

Maria Pia Amato, Department NEUROFARBA, University of Florence, Italy; IRCCS Fondazione Don Carlo Gnocchi, Florence, Italy; Vincenzo Brescia Morra, Multiple Sclerosis Unit, Policlinico Federico II University Hospital, Naples, Italy; Maria Buccafusca, Unit of Neurology and Neuromuscular Disorders, Department of Clinical and Experimental Medicine, University of Messina, Messina, Italy; Sebastiano Bucello, Ospedale Umberto I, Siracusa, Italy; Vincenzo Busillo, MS Center, Maria SS. Addolorata Hospital, ASL Salerno, Eboli, Italy; Massimiliano Calabrese, Neurology B, Department of Neurosciences, Biomedicine and Movement Sciences, University of Verona, Policlinico “G. B. Rossi” Verona, Italy; Elena Colombo, Multiple Sclerosis Centre, Istituto di Ricovero e Cura a Carattere Scientifico Mondino Foundation, Pavia, Italy; Salvatore Cottone, Azienda di Rilievo Nazionale ed. Alta Specializzazione Ospedali Civico Di Cristina Benfratelli, Palermo, Italy; Vincenzo Dattola, Azienda Ospedaliera Bianchi e Melacrino Morelli, UOC Neurologia, Reggio Calabria, Italy; Maria Di Gregorio, Neurology Unit, University Hospital “San Giovanni Di Dio E Ruggi D’Aragona,” Salerno, Italy; Massimo Filippi, Neurology Unit, Neurorehabilitation Unit and Neurophysiology Service, Istituto di Ricovero e Cura a Carattere Scientifico (IRCCS) San Raffaele Scientific Institute, Milan, Italy, Neuroimaging Research Unit, Division of Neuroscience, IRCCS San Raffaele Scientific Institute, Milan, Italy, Vita-Salute San Raffaele University, Milan, Italy; Antonio Gallo, Department of Advanced Medical and Surgical Sciences, University of Campania “Luigi Vanvitelli,” Naples, Italy; Mameli Gianfranco, Neurology Unit, AOU Sassari, Sassari, Italy; Luigi Grimaldi, UOC Neurologia and Centro Sclerosi Multipla, Fondazione Istituto G. Giglio, Cefalù, Italy; Matilde Inglese, University of Genoa, Istituto di Ricovero e Cura a Carattere Scientifico (IRCCS) Ospedale Policlinico San Martino, Genoa, Italy; Alessandra Lugaresi, Institute of Neurological Sciences of Bologna, Bologna, Italy; Department of Biomedical and Neuromotor Sciences - University of Bologna, Bologna, Italy; Giacomo Lus, Second Division of Neurology, Department of Advanced Medical and Surgery Science, University of Campania Luigi Vanvitelli, Naples, Italy; Girolama Alessandra Marfia, Multiple Sclerosis Clinical and Research Unit, Fondazione Policlinico Tor Vergata, Department of Systems Medicine, University Tor Vergata, Rome, Italy; Livia Pasquali, Department of Clinical and Experimental Medicine, Clinical Neurology, University of Pisa, Pisa, Italy; Paolo Ragonese, UOC Neurologia and Centro Sclerosi Multipla, Fondazione Istituto G. Giglio, Cefalù, Italy; Marco Rovaris, Fondazione Don Carlo Gnocchi Istituto di Ricovero e Cura a Carattere Scientifico, Milan, Italy; Augusto Maria Rini, Ospedale A. Perrino, Brindisi, Italy; Edoardo Sessa, Centro Neurolesi Bonino Pulejo Istituto di Ricovero e Cura a Carattere Scientifico, Messina, Italy; Paola Valentino, Institute of Neurology, Magna Græcia University of Catanzaro, Catanzaro, Italy.
