# Supplementary material for: Impact of cladribine tablets on PROs in patients with MS: insights from the 1st interim analysis of the CLADFIT-MS study
Source: Front Neurol. 2026 Apr 10;17:1765153. doi: 10.3389/fneur.2026.1765153 (PMC13107940; doi:10.3389/fneur.2026.1765153)
Supplement: Supplementary file 1 [file Table_1.DOCX]

**Supplementary Table 1: Inclusion and exclusion criteria.**

| **Inclusion/Exclusion Criteria** |
| --- |
| **Inclusion Criteria**   1. Subjects ≥ 18 years old; 2. Female and male subjects with highly-active MS switching to cladribine tablets as their first second-line treatment in routine clinical practice, following the SmPC specifications (high activity disease defined as subjects with 1 relapse in the previous year and at least 1 T1 Gd+ lesion, or 9 or more T2 lesions while on therapy with other DMDs, or subjects with 2 or more relapses in the previous year, whether on DMD treatment or not); 3. Subjects providing written informed consent to participate and release their personal data for the scientific purposes of the study. |
| **Exclusion Criteria**   1. Subjects who, at the discretion of the investigator, are not able to provide reliable information for the study or will be probably lost to follow-up during the first months of the study; 2. Contraindications to use of cladribine tablets according to the SmPC; 3. Subjects on a DMD with a washout period of over 12 weeks, according to the DMD’s SmPC; 4. Subjects previously treated with a second-line MS therapy; 5. Clinically relevant anxiety and depression disorders, which at the discretion of the investigator, represent an impeding factor for the study participation; 6. Subjects participating in interventional clinical trials. |
| Abbreviations: DMDs: Disease Modifying Drugs; Gd+: Gadolinium-enhancing; MS: Multiple Sclerosis; SmPC: Summary of Product Characteristics. |
