# Supplementary material for: Impact of cladribine tablets on PROs in patients with MS: insights from the 1st interim analysis of the CLADFIT-MS study
Source: Front Neurol. 2026 Apr 10;17:1765153. doi: 10.3389/fneur.2026.1765153 (PMC13107940; doi:10.3389/fneur.2026.1765153)
Supplement: Supplementary file 2 [file Table_2.DOCX]

**Supplementary Table 2: Disease history - Details on relapses.**

|  | **N=190** |
| --- | --- |
| **Maximal EDSS at the time of the relapse** |  |
| N (%) | 83 (43.7) |
| Missing, n (%) | 107 (56.3) |
| Mean (SD) | 2.4 (0.99) |
| Median | 2.0 |
| Q1; Q3 | 2.0, 2.5 |
| **Major systems affected at the time of the relapse, n (%)** |  |
| Pyramidal function | 47 (24.7) |
| Cerebellar function | 15 (7.9) |
| Brain stem function | 18 (9.5) |
| Sensory function | 102 (53.7) |
| Bowel and bladder function | 14 (7.4) |
| Visual (optic) function | 24 (12.6) |
| Cerebral (mental) function | 10 (5.3) |
| **The worst severity by ADL history, n (%)** |  |
| Mild | 81 (42.6) |
| Moderate | 35 (18.4) |
| Severe | 5 (2.6) |
| Unknown | 69 (36.3) |
| **Use of steroid treatment for MS relapse, n (%)** |  |
| Yes | 95 (50.0) |
| No | 95 (50.0) |
| **Hospitalization due to MS relapse, n (%)** |  |
| Yes | 5 (2.6) |
| No | 185 (97.4) |
| **Total number of days of hospitalization** |  |
| N (%) | 4 (2.1) |
| Missing, n (%) | 1 (0.5) |
| Mean (SD) | 21.8 (33.51) |
| Median | 5.5 |
| Q1; Q3 | 4.5, 39.0 |
| Abbreviations: ADL: Activities of Daily Living; EDSS: Expanded Disability Status Scale; MS: Multiple Sclerosis; Q1, Q3: Interquartile range; SD: Standard Deviation. | |
