# Supplementary material for: Impact of cladribine tablets on PROs in patients with MS: insights from the 1st interim analysis of the CLADFIT-MS study
Source: Front Neurol. 2026 Apr 10;17:1765153. doi: 10.3389/fneur.2026.1765153 (PMC13107940; doi:10.3389/fneur.2026.1765153)
Supplement: Supplementary file 3 [file Table_3.DOCX]

**Supplementary Table 3: Summary of MSIS-29 physical domain scores.**

| **Visit** | **n (% of N=190)** | **Missing forms, n (%)** | **Mean (SD)** | **95% CI (LCL; UCL)** | **Median** | **Q1; Q3** | **Min; Max** |
| --- | --- | --- | --- | --- | --- | --- | --- |
| **Baseline -**  **Observed values** | 186 (97.9) | 4 (2.1) | 13.4 (14.21) | 11.3; 15.4 | 8.8 | 2.5; 20.0 | 0.0; 66.3 |
| **Baseline -**  **Patients with ≥1 missing item** | 1 (0.5) | - | 9.0 (N/A*) | - | 9.0 | 9; 9 | 9; 9 |
| **Week 52 -**  **Observed values** | 161 (84.7) | 29 (15.3) | 12.8 (15.68) | 10.4; 15.3 | 6.3 | 1.3; 17.5 | 0.0; 70.0 |
| **Week 52 -**  **Patients with ≥1 missing item** | 0 (0.0) | - | - | - | - | - | - |
| * Only one patient had missing items at baseline; therefore, the SD was not defined.  Abbreviations: CI: Confidence Interval; LCL: Lower Confidence Limit; Q1; Q3: Interquartile Range. SD: Standard Deviation; UCL: Upper Confidence Limit. | | | | | | | |
