# Supplementary material for: Impact of cladribine tablets on PROs in patients with MS: insights from the 1st interim analysis of the CLADFIT-MS study
Source: Front Neurol. 2026 Apr 10;17:1765153. doi: 10.3389/fneur.2026.1765153 (PMC13107940; doi:10.3389/fneur.2026.1765153)
Supplement: Supplementary file 4 [file Table_4.DOCX]

**Supplementary Table 4: Changes in MSIS-29 physical scores considering evaluations from wearable activity trackers - Univariate mixed model analysis.**

|  | **Estimate (SE)** | **Degrees of freedom** | **p-value** | **95% CI** |
| --- | --- | --- | --- | --- |
| **Range of movement (number of steps)** | | | | |
| **Baseline** | -0.1 (0.04) | 101.1 | 0.0425 | -0.17; -0.00 |
| **Range of movement** | -0.0 (<0.01) | 107.3 | 0.3621 | -0.00; 0.00 |
| **Baseline*Range of movement** | -0.0 (<0.01) | 106.4 | 0.2875 | -0.00; 0.00 |
| **Burned calories (kcal)** | | | | |
| **Baseline** | -0.1 (0.04) | 101.0 | 0.0544 | -0.17; 0.00 |
| **Burned calories** | -0.0 (<0.01) | 110.5 | 0.1828 | -0.00; 0.00 |
| **Baseline*Burned calories** | 0.0 (<0.01) | 105.7 | 0.5974 | -0.00; 0.00 |
| **Heart rate (beats per minute)** | | | | |
| **Baseline** | -0.1 (0.04) | 100.9 | 0.0684 | -0.17; 0.01 |
| **Heart rate** | 0.0 (0.09) | 106.7 | 0.8862 | -0.17; 0.19 |
| **Baseline*Heart rate** | -0.0 (0.01) | 107.9 | 0.9245 | -0.01; 0.01 |
| **Sleeping time (hours)** | | | | |
| **Baseline** | -0.0 (0.04) | 90.9 | 0.6150 | -0.11; 0.06 |
| **Sleeping time** | -0.0 (0.15) | 105.4 | 0.9483 | -0.30; 0.29 |
| **Baseline*Sleeping time** | -0.0 (0.01) | 105.2 | 0.3432 | -0.02; 0.01 |
| Abbreviations: CI : Confidence Interval; SE: Standard Error. | | | | |
