# Supplementary material for: Impact of cladribine tablets on PROs in patients with MS: insights from the 1st interim analysis of the CLADFIT-MS study
Source: Front Neurol. 2026 Apr 10;17:1765153. doi: 10.3389/fneur.2026.1765153 (PMC13107940; doi:10.3389/fneur.2026.1765153)
Supplement: Supplementary file 5 [file Table_5.DOCX]

**Supplementary Table 5: Fitbit^®^ parameters*.**

|  | **Baseline** | **Week 52** | **Change**  **from baseline**** |
| --- | --- | --- | --- |
| **Range of movement (number of steps)** | | | |
| **N (%)** | 160 (84.2) | 125 (65.8) | 117 (61.6) |
| **Missing, N (%)** | 30 (15.8) | 65 (34.2) | 73 (38.4) |
| **Mean (SD)** | 16,295.4 (10,835.76) | 16,398.6 (11,426.59) | -117.3 (11,257.41) |
| **Median** | 16,132.0 | 15,774.0 | -541.0 |
| **Q1; Q3** | 9,576.5, 22,044.0 | 7,062.0, 21,980.0 | -5,341.0, 3,651.0 |
| **Walking distance (meters)** | | | |
| **N (%)** | 37 (19.5) | 35 (18.4) | 14 (7.4) |
| **Missing, N (%)** | 153 (80.5) | 155 (81.6) | 176 (92.6) |
| **Mean (SD)** | 2,095.8 (1,409.47) | 2,392.2 (1,633.71) | 634.6 (2,265.31) |
| **Median** | 1,689.0 | 1,925.1 | 550.6 |
| **Q1; Q3** | 1,193.8, 2,246.6 | 1,112.7, 2,826.3 | -1,004.5, 1,557.7 |
| **Walking speed (m/s)** | | | |
| **N (%)** | 37 (19.5) | 35 (18.4) | 14 (7.4) |
| **Missing, N (%)** | 153 (80.5) | 155 (81.6) | 176 (92.6) |
| **Mean (SD)** | 0.9 (0.15) | 1.0 (0.17) | 0.0 (0.19) |
| **Median** | 0.9 | 1.0 | 0.0 |
| **Q1; Q3** | 0.8, 1.0 | 0.8, 1.1 | -0.1, 0.2 |
| **Burned calories (kcal)** | | | |
| **N (%)** | 160 (84.2) | 125 (65.8) | 117 (61.6) |
| **Missing, N (%)** | 30 (15.8) | 65 (34.2) | 73 (38.4) |
| **Mean (SD)** | 4,164.2 (2,038.12) | 3,980 (2,058.53) | -207.1 (2,043.90) |
| **Median** | 4,105.0 | 4,012.0 | -290.0 |
| **Q1; Q3** | 3,279.0, 5,134.5 | 3,020.0, 5,125.0 | -975.0, 479.0 |
| **Heart rate (beats per minute)** | | | |
| **N (%)** | 160 (84.2) | 125 (65.8) | 117 (61.6) |
| **Missing, N (%)** | 30 (15.8) | 65 (34.2) | 73 (38.4) |
| **Mean (SD)** | 80.2 (7.47) | 79.8 (8.24) | -1.2 (10.04) |
| **Median** | 79.0 | 79.0 | -1.0 |
| **Q1; Q3** | 75.2, 84.1 | 74.3, 83.2 | -5.0, 3.0 |
| **Sleeping time (hours)** | | | |
| **N (%)** | 161 (84.7) | 121 (63.7) | 114 (60.0) |
| **Missing, N (%)** | 29 (15.3) | 69 (36.3) | 76 (40.0) |
| **Mean (SD)** | 11.0 (5.57) | 10.3 (5.64) | -0.5 (6.18) |
| **Median** | 12.6 | 11.7 | -0.3 |
| **Q1; Q3** | 8.1, 14.6 | 6.9, 14.7 | -4.1, 1.8 |
| * Evaluation of data from Fitbit® wearable activity trackers collected over two consecutive days at baseline, Week 52, and Week 104.  ** Change from baseline included only those patients with both a baseline and Week 52 value.  Abbreviations: Q1, Q3: Interquartile Range; SD: Standard Deviation. | | | |
