# Supplementary material for: Impact of cladribine tablets on PROs in patients with MS: insights from the 1st interim analysis of the CLADFIT-MS study
Source: Front Neurol. 2026 Apr 10;17:1765153. doi: 10.3389/fneur.2026.1765153 (PMC13107940; doi:10.3389/fneur.2026.1765153)
Supplement: Supplementary file 6 [file Table_6.DOCX]

**Supplementary Table 6: Summary of Fitbit^®^ devices evaluations.**

| **Parameter (unit)** | **Timepoint** | **n (% of N=190)** | **Missing, n (%)** | **Mean (SD)** | **Median** | **Q1; Q3** | **Min; Max** |
| --- | --- | --- | --- | --- | --- | --- | --- |
| **Range of movement (number of steps)** | Baseline | 160 (84.2) | 30 (15.8) | 16,295 (10,836) | 16,132 | 9,577; 22,044 | 0; 61,426 |
|  | Week 52 | 125 (65.8) | 65 (34.2) | 16,399 (11,427) | 15,774 | 7,062; 21,980 | 0; 53,892 |
|  | Δ Week 52 vs baseline | 117 (61.6) | 73 (38.4) | -177 (11,257) | 541 | -5,341; 3,651 | -42,022; 30,139 |
| **Walking distance (meters)** | Baseline | 37 (19.5) | 153 (80.5) | 2,096 (1,409) | 1,689 | 1,194; 2,247 | 711; 6,115 |
|  | Week 52 | 35 (18.4) | 155 (81.6) | 2,392 (1,634) | 1,925 | 1,113; 2,826 | 34; 6,067 |
|  | Δ Week 52 vs baseline | 14 (7.4) | 176 (92.6) | 635 (2,265) | 551 | -1,005; 1,558 | -3,636; 4,788 |
| **Walking speed (m/s)** | Baseline | 37 (19.5) | 153 (80.5) | 0.90 (0.15) | 0.90 | 0.80; 1.00 | 0.50; 1.10 |
|  | Week 52 | 35 (18.4) | 155 (81.6) | 1.00 (0.17) | 1.00 | 0.80; 1.10 | 0.60; 1.30 |
|  | Δ Week 52 vs baseline | 14 (7.4) | 176 (92.6) | 0.00 (0.19) | 0.00 | -0.10; 0.20 | -0.30; 0.30 |
| **Burned calories (kcal)** | Baseline | 160 (84.2) | 30 (15.8) | 4,164 (2,038) | 4,105 | 3,279; 5,135 | 28; 9,101 |
|  | Week 52 | 125 (65.8) | 65 (34.2) | 3,980 (2,059) | 4,012 | 3,020; 5,125 | 0; 10,200 |
|  | Δ Week 52 vs baseline | 117 (61.6) | 73 (38.4) | -207 (2,044) | -290 | -975; 479 | -7,627; 6,072 |
| **Heart rate (beats per minute)** | Baseline | 160 (84.2) | 30 (15.8) | 80.2 (7.47) | 79.0 | 75.2; 84.1 | 63.5; 108.6 |
|  | Week 52 | 125 (65.8) | 65 (34.2) | 79.8 (8.24) | 79.0 | 74.3; 83.2 | 60.6; 121.0 |
|  | Δ Week 52 vs baseline | 117 (61.6) | 73 (38.4) | -1.2 (10.0) | -1.0 | -5.0; 3.0 | -32.7; 44.3 |
| **Sleeping time (hours)** | Baseline | 161 (84.7) | 29 (15.3) | 11.0 (5.57) | 12.6 | 8.1; 14.6 | 0; 24.4 |
|  | Week 52 | 121 (63.7) | 69 (36.3) | 10.3 (5.64) | 11.7 | 6.9; 14.7 | 0; 27.7 |
|  | Δ Week 52 vs baseline | 114 (60.0) | 76 (40.0) | -0.5 (6.18) | -0.3 | -4.1; 1.8 | -16.7; 16.4 |
| Abbreviations: Q1; Q3: Interquartile Range; SD: Standard Deviation. | | | | | | | |
