# Supplementary material for: Impact of cladribine tablets on PROs in patients with MS: insights from the 1st interim analysis of the CLADFIT-MS study
Source: Front Neurol. 2026 Apr 10;17:1765153. doi: 10.3389/fneur.2026.1765153 (PMC13107940; doi:10.3389/fneur.2026.1765153)
Supplement: Supplementary file 7 [file Table_7.DOCX]

**Supplementary Table 7: Effective sample sizes for wearable outcomes.**

| **Parameter** | **Evaluable at baseline** | **Evaluable at Week 52** | **Evaluable for change*** |
| --- | --- | --- | --- |
| **Range of movement (number of steps)** | 160 | 125 | 117 |
| **Walking distance (meters)** | 37 | 35 | 14 |
| **Walking speed**  **(m/s)** | 37 | 35 | 14 |
| **Burned calories**  **(kcal)** | 160 | 125 | 117 |
| **Heart rate**  **(beats per minute)** | 160 | 125 | 117 |
| **Sleeping time**  **(hours)** | 161 | 121 | 114 |
| * Evaluable for change: Patients with both baseline and Week 52 data, i.e., those included in the change from baseline analysis. | | | |
