# Supplementary material for: Impact of cladribine tablets on PROs in patients with MS: insights from the 1st interim analysis of the CLADFIT-MS study
Source: Front Neurol. 2026 Apr 10;17:1765153. doi: 10.3389/fneur.2026.1765153 (PMC13107940; doi:10.3389/fneur.2026.1765153)
Supplement: Supplementary file 8 [file Supplementary_file_1.docx]

Local Ethics Committees:

*Comitato Etico AOU Consorziale Policlinico di Bari, Comitato Etico Campania Sud, Comitato Etico Catania 1, Comitato Etico Catania 2, Comitato Etico della Fondazione A. Gemelli IRCCS, Comitato Etico della regione Liguria, Comitato Etico dell'Univeristà Federico II, Comitato Etico Indipendente di area vasta Emilia centro, Comitato Etico Indipendente della Fondazione PTV Policlinico Tor Vergata, Comitato Etico Indipendente dell'Azienda Ospedaliero Universitaria di Cagliari, Comitato Etico IRCCS Fondazione Don Carlo Gnocchi, Comitato Etico IRCCS Ospedale San Raffaele, Comitato Etico IRCCS Sicilia Centro Neurolesi "Bonino-Pulejo", Comitato Etico Lazio 2, Comitato Etico Messina, Comitato Etico Palermo 1, Comitato Etico Palermo 2, Comitato Etico Pavia, Comitato Etico per la Sperimentazione Clinica (CESC) delle Province di Verona e Rovigo, Comitato Etico Regione Calabria sezione Area Centro, Comitato Etico Regione Calabria sezione Area Sud, Comitato Etico Regione Toscana - Area vasta centro, Comitato Etico Regione Toscana-Area Nord Ovest, Comitato Etico Università Vanvitelli di Napoli, Comitato indipendente di etica medica ASL Brindisi.*
